# Involvement of Apoptosis in Host-Parasite Interactions in the Zebra Mussel

**DOI:** 10.1371/journal.pone.0065822

**Published:** 2013-06-13

**Authors:** Laëtitia Minguez, Nelly Brulé, Bénédicte Sohm, Simon Devin, Laure Giambérini

**Affiliations:** Université de Lorraine, Laboratoire des Interactions, Ecotoxicologie, Biodiversité, Ecosystèmes (LIEBE), CNRS UMR 7146, Metz, France; NIAID, United States of America

## Abstract

The question of whether cell death by apoptosis plays a biological function during infection is key to understanding host-parasite interactions. We investigated the involvement of apoptosis in several host-parasite systems, using zebra mussels *Dreissena polymorpha* as test organisms and their micro- and macroparasites. As a stress response associated with parasitism, heat shock proteins (Hsp) can be induced. In this protein family, Hsp70 are known to be apoptosis inhibitors. Mussels were diagnosed for their respective infections by standard histological methods; apoptosis was detected using the TUNEL methods on paraffin sections and Hsp70 by immunohistochemistry on cryosections. Circulating hemocytes were the main cells observed in apoptosis whereas infected tissues displayed no or few apoptotic cells. Parasitism by intracellular bacteria Rickettsiales-like and the trematode *Bucephalus polymorphus* were associated with the inhibition of apoptosis whereas ciliates *Ophryoglena* spp. or the trematode *Phyllodistomum folium* did not involve significant differences in apoptosis. Even if some parasites were able to modulate apoptosis in zebra mussels, we did not see evidence of any involvement of Hsp70 on this mechanism.

## Introduction

Apoptosis, a form of programmed cell death, was highly conserved evolutionarily and plays a central role in homeostasis and normal tissue development, where the old or damaged cells have to be eliminated. It was defined by characteristic morphological and biochemical features such as cell shrinkage, chromatin condensation and DNA fragmentation [1]. In invertebrates, apoptosis can be induced after exposure to pollutants [2], [3], [4], [5], but also by pathogens or parasites. This process plays a major role in the host defense by preventing the spread of parasites and pathogens [4], [6], [7], [8], [9]. However, apoptosis induction is not systematic and depends on parasite species. Indeed, they have developed the ability to modulate apoptotic signals in cells of their host [10]. Some are able to induce apoptosis to escape the defense systems of the host [6], [9], [11], [12] whereas others, like intracellular parasites, will rather inhibit cell death to establish a sustainable interaction with the host [6], [13], [14], [15], [16].

This cell death process has been widely studied on host-parasite systems with medical and/or economic interests, particularly with vertebrates as hosts (reviewed in [17] and [18]). Studies on bivalves rest upon early research [1], [4], [7], [13], [19], [20]. Nonetheless, aquatic ecosystems are highly dynamic and thus provide a wide variety of biotic and abiotic stress factors for individuals living there [21]. Hosts must be able to defend themselves, and parasites must be able to manipulate host defenses for their establishment in a host. Barcinski & DosReis [22] have shown that regulation of host cell apoptosis is a critical determinant factor in host-parasite interactions.

Living systems have developed a variety of strategies to respond to environmental stress. Among them, heat shock proteins (Hsp) form one of the most ancient defense systems. These proteins play a major role in cytoprotection by acting as molecular chaperones, i.e. helping in the refolding of misfolded proteins and assisting in their elimination if they become irreversibly damaged [21]. These proteins are constitutively expressed in cells to maintain these cellular processes but can also be induced in response to a variety of biotic and abiotic stressors. An increased expression of Hsp may be associated with apoptosis resistance. Proteins of this family are classified by their molecular weight and for example Hsp70 (protein of 70 kDa) has been shown able to act as apoptosis inhibitor [23].

Due to their immobility, sessile organisms like bivalves represent good biological models. Because bivalves are highly susceptible to their surrounding environment, it could be suggested that a strong apoptotic process may be necessary to ensure body homeostasis [24]. In the present study, we chose zebra mussels *Dreissena polymorpha* as test organisms. Because of their ubiquitous distribution and high contaminant uptake, these freshwater bivalves are commonly used in ecotoxicological studies, and they can be the host of more than 40 parasite species, from micro- to macroparasites [25], [26], [27], [28]. Herein, we investigated the interactions between zebra mussels and the most observed parasite species in our region, i.e. intracellular bacteria Rickettsiales-like (RLOs), extracellular ciliates *Ophryoglena* spp. in the digestive gland, and extracellular cysts of trematodes *Phyllodistomum folium* in gills and *Bucephalus polymorphus* in gonads. Our previous studies have shown that these parasites induced some physiological disturbances more or less important for their host [29], [30], [31], [32]. However, to better understand these host-parasite systems, it is important to go into the finer mechanisms underlying the establishment of the parasite. The parasite-induced modulation of apoptosis could play a major role. Thus, in this paper we focused on apoptosis of host cells in several zebra mussel - parasite complexes. We also looked at stress response associated with parasitism (i.e. increased expression of Hsp70) since this protein is known to act as an apoptosis inhibitor.

## Materials and Methods

No specific permits were required for the described field studies because we work on public area. The field studies did not involve endangered or protected species.

### Zebra Mussel Sampling and Tissue Preparation

Zebra mussels were randomly handpicked from under rocks on the shores of three rivers of the northern half of France, depending on the investigated parasites. (1) For ciliates *Ophryoglena* spp. (Oph) and intracellular bacteria Rickettsiales-like organisms (RLO), mussels (N = 63) were sampled in the Moselle River downstream from the wastewater treatment plant of Metz (49°10′46.45″N, 06°11′56.71″E) in May. (2) To obtain enough organisms infected by the trematodes *Phyllodistomum folium* or *Bucephalus polymorphus* a sampling effort was realized. Zebra mussels (N = 564) were collected in the Meuse River at Troussey in March for *P. folium* (48°42′13.89″N, 5°42′02.99″E) and (3) in the Vilaine River at Langon (47°42′56.95″N, 1°50′13.41″W) in August for *B. polymorphus* (N = 905). The shell length range of mussels from the Moselle River and Langon was 15–21 mm, and 23–37 mm for organisms from Troussey. All the sampled mussels were adult.

A part of the digestive gland was excised from each organism and prepared as described in Giambérini and Cajaraville [33] for Hsp70 immunohistochemistry assay. Briefly, the freshly removed tissues were cryoprotected, embedded in a synthetic resin and frozen in nitrogen vapor. Sections of 8 µm thickness were cut in a Leica CM3000 cryostat (Leica Instruments GmbH, Germany), collected on Superfrost slides, and stored at −80°C until required for staining. All the remaining body was fixed for 48 h in Bouin’s Fixative, rinsed in water and embedded in paraffin after dehydration in graded series of ethanol–Roti-Histol. The tissue sections (5 µm thick, 2 glass slides) were used for parasite identification and gonadal index determination after Gill II hematoxylin/eosin staining, or for detecting apoptosis (see below). On each section, all the main tissues were observed, i.e. gills, gonads, digestive gland and connective tissue.

### Parasite Identification and Gonadal Index Determination

The procedure for parasite inventory is described by Minguez et al. [29]. Briefly 30–40 sections per zebra mussel were studied microscopically for presence of parasites. After this inventory several fourteen experimental groups were formed according to parasite species or association of parasites (table 1).

**Table 1:** Number of zebra mussels in each experimental group.

		N		N
Males :	Non-infected	8	Non-infected	16
	Oph	5	*P. folium*	16
	RLO	8		
	Oph-RLO	4		
Females :	Non-infected	8	Non-infected	9
	Oph	8	*P. folium*	9
	RLO	8		
	Oph-RLO	3		

For the study of *B. polymorphus*, 12 males and 12 females formed the non-infected group. 24 mussels were infected (castration).

Gonad maturity was assessed by microscopic observation of slides, through determination of a mean gonadal index (GI) for each experimental group as described by Tourari et al. [34]. Zebra mussels were classified in one of six successive stages of gonad maturation. An arbitrary score from 0 to 5 was attributed to each stage, and the following formula was used to calculate gonadal index: GI = (∑n_i_*s_i_)/N where n_i_ is the number of individuals in each stage, s_i_ the score of the stage and N the total number of individuals.

### DNA Fragmentation Evaluation

Apoptosis was examined using the TUNEL methods. The FragEL™ DNA Fragmentation Detection Kit (Merck Biosciences, Cat. No QIA39) was applied following the manufacturer’s instructions: sections were deparaffinised and rehydrated in a descending Roti-Histol–Ethanol series before being brought to Tris-buffered saline (TBS). The sections were then pre-treated with Proteinase K (20 µg.mL^−1^, 15 min, at room temperature) in Tris buffer (10 mM, pH 8). They were quenched in 3% hydrogen peroxide in phosphate-buffered saline (PBS) for 5 min, rinsed in distilled water, and treated with equilibration buffer (1h30, at room temperature). Sections were then incubated with TdT labeling reaction mix (1 h, moist chamber, 37°C). Sections were then rinsed in TBS, counterstained in Gill II hematoxylin and mounted under a glass coverslip using Fluorescein-FragEL™ mounting media. Negative controls were made by substituting TdT with water and positive controls by using DNAse I at 1 µg.µL^−1^ in TBS-1mM MgSO_4_ solution for 20 min at room temperature.

Stained cells were counted on three histological sections (separated by at least 15 µm) per zebra mussel in a fluorescence microscope (standard fluorescein filter, 465–495 nm), and the mean number of apoptotic cells was reported to the section area estimated using Cell* software (Olympus) and a graphic tablet (Wacom® Cintiq® 21× pen display).

### Detection of Hsp70 Expression by Immunohistochemistry

Sections were fixed for 7 min in 4% paraformaldehyde dissolved in PBS at pH 7.4. Sections were then sequentially rinsed once in phosphate buffer (0.1 M, pH 7.4), once in distilled water, thrice in PBS-0.2% Tween 20 and then blocked for 10 min in PBS-0.2% Triton X-100 containing 0.02% normal horse serum (Vector laboratories, Burlingame, CA, USA). Sections were then incubated overnight at room temperature with primary antibody, a mouse affinity-purified monoclonal anti-70 Kd Hsp (Enzo Life Sciences, Cat. No ADI-SPA-810, dilution 1∶50000 in PBS-Triton containing horse serum). The sections were rinsed thrice in PBS-Tween 20, incubated in 3% hydrogen peroxide – distilled water for 15 min. to quench endogenous peroxydase and rinsed again in PBS-Tween 20 before incubation with secondary antibody for 2 h at room temperature (biotinylated anti-mouse antibody, dilution 1∶200 in serum/PBS-Triton mixture, Vector). Sections were rinsed thrice in the latter medium and covered with the ABC reagent (Vectastain Kit, Vector) for 1 h at room temperature. Sections were rinsed thrice in PBS-Tween 20 and incubated for 3 min in a mixture of 0.02% buffer stock solution, 0.04% diaminobenzidine stock solution and 0.02% hydrogen peroxide in water (DAB substrate kit for peroxydase, Vector). Thereafter, sections were dehydrated in Roti-Histol and coverslipped. In addition, as Hsp 70-positive samples, zebra mussels were treated with a heat shock at 20°C for 6 h and the Hsp70 revelation followed the above protocol. A negative control was also carried out as follows: sections were processed as above except that they were incubated with normal horse serum instead of primary antibody solution in every staining series. An immunolabelling was considered as positive when the staining intensity was greater than the background observed in negative control. The specificity of the monoclonal anti-70 Kd Hsp (primary antibody) for zebra mussel was confirmed by flow cytometry (File S1 and Figure S1).

The Hsp 70 signal was quantified by image analysis (Cell*, Olympus) using a Sony DP 50 colour video camera connected to an Olympus BX 41 microscope with a 100× objective. Eight fields of view were randomly analysed on one section per individual (total sampling area per organism: 101094 µm^2^). Only areas belonging to digestive tissues were considered. The interval of brown shades, corresponding to the reaction, was defined and the surface density occupied by Hsp 70 (Sv_Hsp70_), a stereological parameter, was calculated (Sv_Hsp70_ = S_HSP70_/V_C_, where S = surface, V = Volume, C = digestive cell cytoplasm).

### Data Analysis

Statistical analyses were undertaken with STATISTICA software version 7.1, (Statsoft, USA). P-values less than 0.05 were considered statistically significant. For the study of apoptosis, differences between experimental groups for a same host gender were evaluated using one-way ANOVA followed by Duncan’s post hoc test for groups used in microparasite study cases or a t-test for *P. folium* and *B. polymorphus* studies. These analyses were performed after testing for normality and variance homogeneity of the data. The non-parametric Kruskal-Wallis test was used to evaluate the effect of infection on Hsp70 expression.

## Results

### Apoptosis and Parasite Infection

Circulating hemocytes, especially near the digestive gland, were observed performing apoptosis in both non-infected and infected zebra mussels. The target organs of the different studied parasite species, i.e. digestive gland for microparasites or gills for *P. folium* showed no or few stained cells. No apoptotic cells could be observed in gonads of mussels infected by *B. polymorphus* since the entire organ was replaced by parasite sporocysts.

**Figure 1:**
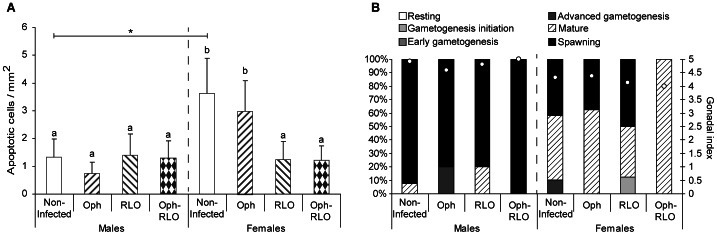
Density of apoptotic cells in zebra mussels infected or not by microparasites and their gonadal development stage. (A) Mean number of apoptotic cells per mm^2^ of tissue in non-infected zebra mussels, infected by ciliates *Ophryoglena* spp. (Oph), or by intracellular bacteria Rickettsiales-like organisms (RLO), or co-infected by these two parasites (Oph-RLO) (+ S.D.). (B) Percentages of mussels at each gamete development stage and gonadal index values (white dots). The host gender was also taken into account. Significant differences between groups of a same gender are indicated by different letters. The star indicates a significant difference between non-infected organisms (one-way ANOVA, Duncan’s post hoc test).

As our previous works on the zebra mussel – their parasite system highlighted gender-specific responses to parasitism [29], [30], [31], the host gender was also taken into account. The infection by microparasites did not cause the same effect on the apoptosis process as regards male or female zebra mussels (Figure 1A). The mean density was two times higher in non-infected females than in males (ANOVA, p = 0.008). The infection in males had no effect on the apoptosis process whereas in females, the presence of RLOs, in single infections or in co-infections, was associated with a significant decrease of apoptotic cell number (ANOVA, p<0.05). Ciliates *Ophryoglena* spp. did not affect apoptosis since no significant differences were observed between non-infected and infected females. Looking at the sexual maturity level of the different experimental groups (Figure 1B), females displayed a slight delay in gonad development, i.e. most of the female mussels were in the mature stage, whereas males were already in spawning stage.

**Figure 2:**
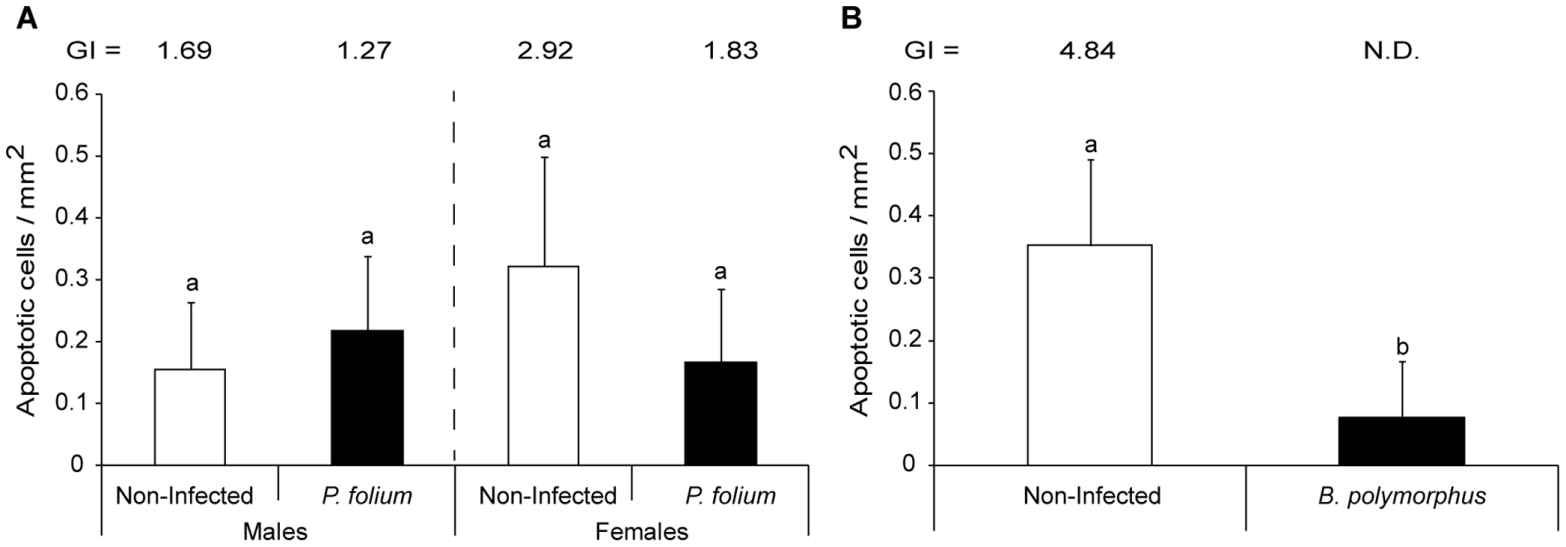
Density of apoptotic cells in zebra mussels infected or not by macroparasites. (A) Mean number of apoptotic cells per mm^2^ of tissue in non-infected zebra mussels or infected by the trematode *Phyllodistomum folium* (+ S.D.). The Gonadal index value is also indicated for each group. (B) Mean number of apoptotic cells per mm^2^ of tissue in non-infected zebra mussels or infected by the trematode *Bucephalus polymorphus* (+ S.D.). The Gonadal index value is only indicated for the non-infected group since it cannot be calculated for the infected one (i.e. castration). Different letters indicate significant differences between groups (t-test).

The host gender in *P. folium* experimental groups (i.e. non- and infected organisms) tended also to induce apoptosis differentially (p = 0.18 but strong intra-group variability), with non-infected females showing two times more apoptotic cells than males, these last being less sexually developed (Figure 2A). For males, the infection by *P. folium* did not involve variation in the apoptosis whereas infected females tended to display half as many apoptotic cells as their non-infected congeners. The infection by *B. polymorphus* induced a significant decrease of apoptosis (t-test, p<0.001) (Figure 2B).

### Expression of Hsp70

**Figure 3:**
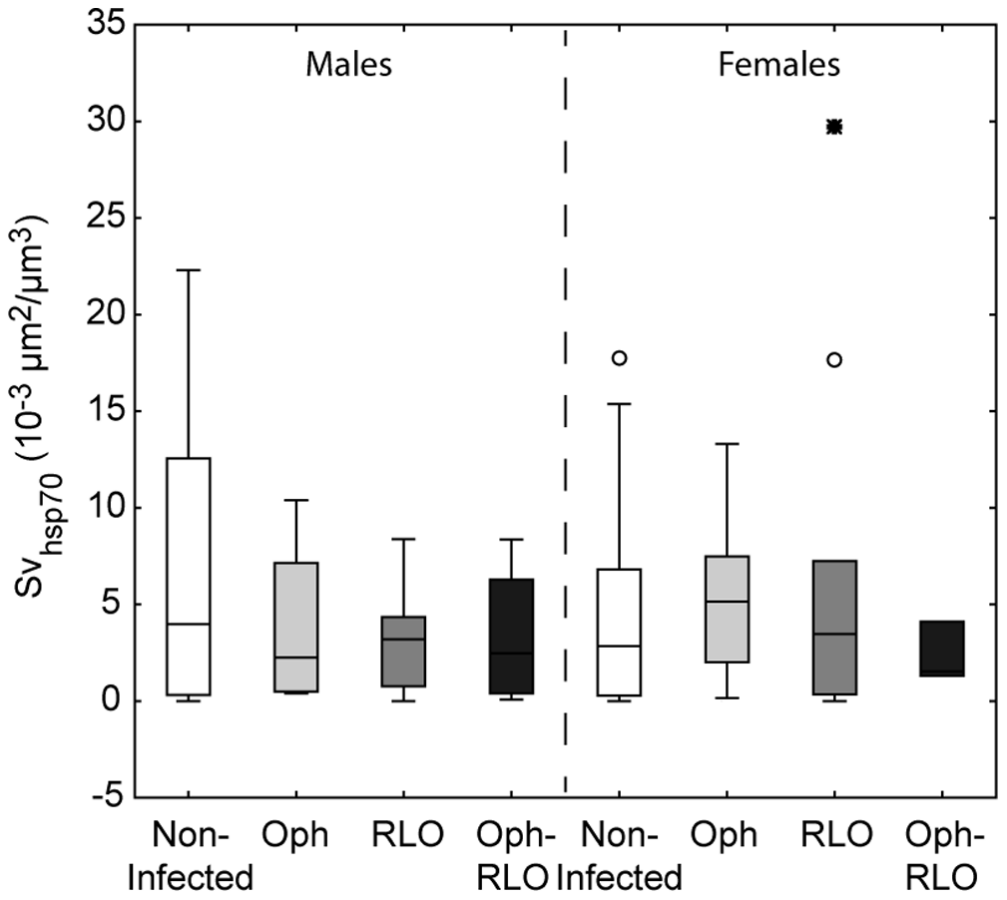
Hsp70 expression in zebra mussels infected or not by microparasites. Hsp 70 expression (Surface density, Sv) in each experimental group of the microparasite study. Both host sex and parasite species (Ophryoglena spp., Oph; Rickettsiales-like organisms, RLOs; co-infections, Oph-RLO) were taken into account. The center line shows the median, 25^th^-75^th^ percentile (within box), minimum/maximum value (error bar). The small circles represent atypical values and the stars the extreme values.

**Figure 4:**
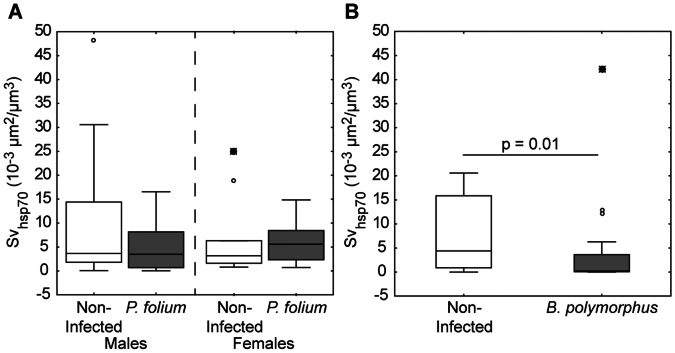
Hsp70 expression in zebra mussels infected or not by macroparasites. Hsp 70 expression (Surface density, Sv) in each experimental group of the macroparasite studies. (A) Comparison of non-infected vs *P. folium*-infected zebra mussels, males or females. (B) Comparison of non-infected vs *B. polymorphus*-infected zebra mussels. Center lines show the median, 25^th^-75^th^ percentile (within box), minimum/maximum value (error bar). The small circles represent atypical values and the stars the extreme values.

In all the studied experimental groups, the immunohistochemical staining was observed in the connective tissues and in the cytoplasm of cells located in the basal part of digestive tubules. The infection by microparasites did not induce variations in Hsp 70 expression in male or female zebra mussels (Figure 3). However, we can note an important intra-group variability. The same was observed for *P. folium* groups with no significant variations of Hsp 70 expression with the infection (Figure 4A). On the contrary, *B. polymorphus* involved a significant decrease of these proteins in the digestive gland (Kruskal-Wallis, p = 0.01) (Figure 4B).

## Discussion

Programmed cell death leading to apoptosis has emerged as a potentially important player in immune defense and in host-parasite relationships. This process has been well studied for vertebrate hosts (reviewed in [17]) and only a few publications focused on molluscs, essentially gastropods and marine bivalves ([35], [36] and reviewed in [1]). On *D. polymorpha*, our team has worked on the physiological modifications linked with parasitism in a context of environmental disturbances [29], [30], [31], [32]. Nonetheless, to better understand the system zebra mussel - its parasites, it seemed interesting to have a more mechanistic approach of the host-parasite relationship, for example, by focusing on apoptosis. Herein, apoptotic cells were revealed by TUNEL assay.

In the present study, apoptosis was only observed in circulating hemocytes. The immune system, due to its role in the defense against chemical and biological foreign particles, must continuously renew its cell stock. Apoptosis enables the adequate clearance of damaged, senescent and infected cells without inflammation [24]. Like for all invertebrates, the immune system of molluscs is based on innate mechanisms where cellular and humoral processes together ensure cytotoxic and antimicrobial functions [37]. Hemocytes play the prominent role in parasite and pathogen elimination. The immune response is complemented by the generation of highly reactive oxygen metabolites (ROS) [38]. An important activity of the immune system can involve a membrane destabilization of hemocytes and eventually apoptosis [39]. Thus, this oxidative stress could explain the apoptosis induction observed in our study. Moreover, according to Sunila & LaBanca [35], apoptosis of hemocytes in the connective tissue may be the mechanism of recycling old hemocytes.

We also noted that target organs of microparasites and *P. folium*, i.e. the digestive gland and gills respectively, displayed none or few apoptotic cells. However, the natural response of infected cells is to commit apoptosis and thus prevent the parasite’s multiplying and spreading [40]. For the digestive gland, a delay in the response of the different organs faced with infection can be hypothesized. Indeed, both ciliates *Ophryoglena* spp. and RLOs displayed low infection intensities with on average 1 ciliate or 3 bacterial inclusions per infected mussels. The individuals would be in an early stage of infection for which the immune system should respond more quickly than the other biological compartments. However, for *P. folium* infection, this assumption is no longer valid, since almost the entire gills were covered with parasite sporocysts, corresponding to an advanced infection stage and few gill cells were apoptotic. In this case, it is more likely that gills displayed higher cell turnover rates. The gill is one of the most active tissues where ROS production is assumed to be high [41]. Moreover, *P. folium* induces gill deformations leading to inflammatory processes [42]. So, a higher cell turnover would compensate for cell injuries, as observed by de Oliveira David et al. [43] in shellfish *Mytella falcata* under stress conditions like pollution.

Microparasites had different effects on zebra mussel immune responses. *Ophryoglena* spp. did not disturb the apoptosis process of hemocytes since non-infected and infected individuals displayed the same mean density of apoptotic cells, whereas RLOs would suppress apoptosis, only for females. More and more studies highlighted the ability of bacteria to modulate the apoptotic cascade in host cells [6], [44], [45], [46], [47]. This kind of response is well known in the bacteria of the Rickettsiales order, and was considered as an adaptive strategy to prolong their life span and proliferation time [36]. The inhibition of apoptosis would be induced by bacterial lipopolysaccharides (LPS) which activate the pathway of the kappa B nuclear factor (NF-κB) playing a role in apoptosis inhibition. This biological pathway has been identified in vertebrate hosts [48], [49] but remains to be demonstrated for zebra mussels.

Helminths are also known to be masterful immunoregulators [50]. Indeed, to facilitate their survival and replication in their intermediate hosts, trematodes are able to down-regulate host defense responses. This has been particularly well studied in gastropod-trematode systems (reviewed in [51] and [52]) but to our knowledge, no studies focused on zebra mussels. In the present investigation, two parasitic helminths were studied and only the castrating trematode *B. polymorphus* was associated with a significant decrease of apoptotic hemocyte density. This decrease is probably linked only to a decrease of the total hemocytes as it was shown by Da Silva et al. [53] on the system *Perna Perna* – *Bucephalus* sp. Like in the present investigation, no inflammation nor encapsulation responses have been induced. *Bucephalus polymorphus* seemed to be able to evade mussel immune defenses, as already observed for trematodes of the same genus parasitizing mussels and oysters [53]. Moreover, by infecting gonads, and causing little host physiological disturbances, this trematode would increase the life span of the host-parasite complex [30].

Another interesting result is the difference between genders, with non-infected females showing more apoptotic cells than non-infected males. Even if zebra mussels used for the microparasite study were sampled at the same time, most females displayed a slight delay in their sexual development with mature gonads (GI≈4) whereas males were already spawning (GI≈5). During the reproduction cycle of bivalves, catecholamines play a role in gonad maturation, and reach their maximum concentrations when the gonads are mature before decreasing rapidly during spawning [54], [55]. Lacoste et al. [19] have highlighted neuro-immuno-endocrine interactions in the oyster *Crassostrea gigas* where these hormones were able to induce apoptosis of the hemocytes. The same interaction could happen for zebra mussels where females with mature gonads would display higher concentrations of catecholamines than males and so, more apoptotic hemocytes.

Parasites remain one of the inducers of the stress response involving the synthesis of HSPs which protect cells from irreversible damage of inflammatory products such as ROS, and from death (reviewed in [21]). The modulatory effects of HSPs on apoptosis are well documented. Among this protein family, the hsp70 is known to have anti-apoptotic properties [23]. Moreover, Xu and Faisal [56] have suggested that hsp70 has a major role in the host defense mechanisms in zebra mussels. Thus, in our study, the infection by microparasites or trematodes would induce the synthesis of hsp70, particularly in mussels parasitized with RLOs (single or co-infections) since an inhibition of apoptosis has been observed. However, we found no differences between non-infected and organisms infected by microparasites or *P. folium*. It is known that the level and the duration of hsp70 induction depend on the nature of the stress factor. For example, the studies of Singer et al. [57] demonstrated that exposure to platinum group metals involved a hsp70 induction in *D. polymorpha* only after 18 days post-exposure and levels remained elevated for about a week. On the contrary, Xu and Faisal [56], by studying RNA of hemocytes stimulated by bacterial LPS, have shown that the induction was much faster (1 h) and much shorter in duration (a few hours). In our study, even if the infection by microparasites was in an early stage, the induction has probably already occurred and levels returned to their baseline. Concerning zebra mussels infected by *B. polymorphus*, they displayed significantly less hsp70 than non-infected congeners. The production of HSP is costly to the organism, as it requires extensive energy and interferes with normal cell functions [58]. However, the host exploitation strategy of this trematode is to reduce the effect of infection on host survival to ensure its reproduction (i.e. infection of gonads, a non-vital organ, and nutritional resources are redirected to parasite development) [30]. Thus, all processes expensive in energy could hinder the parasite development. Moreover, some trematode, like *Schistosoma mansoni*, are known to release a variety of molecules called excretory-secretory products (ESP) able to interact with some host functions like the immune system (reviewed in [59]). Zahoor et al. [59] showed that host Hsp70 protein levels were attenuated in the snail *Biomphalaria glabrata* hemocytes by the trematode *S. mansoni* ESPs. The response observed in zebra mussels infected by the trematode *B. polymorphus*, i.e. a decrease in Hsp70 levels, could also involved ESP release.

### Conclusion

This first study on apoptosis in the interaction between *D. polymorpha* and its parasites showed that most of them were able to manipulate the host immune system. Especially, the intracellular bacteria RLOs and the trematode *B. polymorphus* were able to inhibit apoptosis. Future research will provide new insights into the apoptotic pathway (e.g. to confirm the use of the NF-κB pathway). At the moment, the highlighting of the involvement of hsp70 in apoptosis remains difficult by *in vivo* studies. Indeed, its induction is transient and laboratory infections are not yet under control. However, *in vitro* methods coupled with molecular techniques could be considered to understand the biological mechanisms underlying the interaction of the zebra mussel and its parasites.

## Supporting Information

Figure S1 **Labeling specificity of Hsp70 antibody on zebra mussel Hsp70 protein.** For histograms representation, the same population (total hemocytes) was gated in size/granularity (FSC/SSC), as shown in (A). (B) Histogram of total hemocytes labeled with isotype control. Most of cells have no fluorescence in FL1. (C) Histogram of total hemocytes labeled with HSP70-FITC antibody. Labeled hemocytes show a strong fluorescence in FL1 compared to the isotype control. This intensity shift highlights antibody specificity. Hemocytes labeling and cytometry analysis were done three independent times.
(TIF)

File S1 **Assessment of the specificity of Hsp70 antibody (clone C92F3A5) on zebra mussel Hsp70 protein.**
(DOCX)
