# Supplementary figures and images for: Involvement of Apoptosis in Host-Parasite Interactions in the Zebra Mussel
Source: PLoS One. 2013 Jun 13;8(6):e65822. doi: 10.1371/journal.pone.0065822 (PMC3681881; doi:10.1371/journal.pone.0065822)

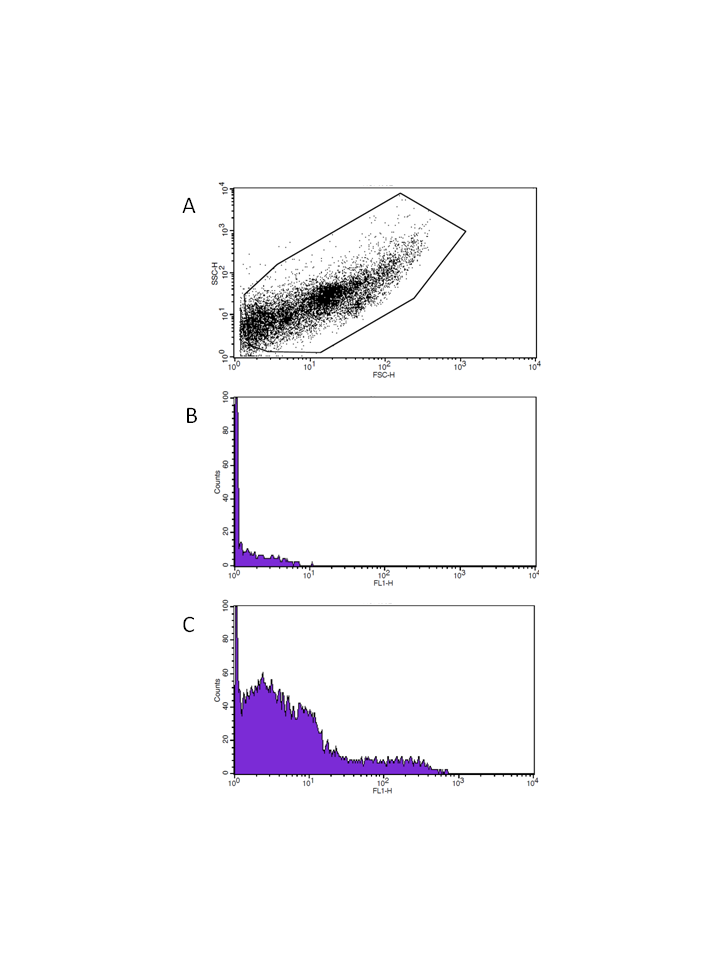

Supplement: Figure S1 — Labeling specificity of Hsp70 antibody on zebra mussel Hsp70 protein. For histograms representation, the same population (total hemocytes) was gated in size/granularity (FSC/SSC), as shown in (A). (B) Histogram of total hemocytes labeled with isotype control. Most of cells have no fluorescence in FL1. (C) Histogram of total hemocytes labeled with HSP70-FITC antibody. Labeled hemocytes show a strong fluorescence in FL1 compared to the isotype control. This intensity shift highlights antibody specificity. Hemocytes labeling and cytometry analysis were done three independent times. (TIF) [file pone.0065822.s001.tif]
