# Supplementary material for: Involvement of Apoptosis in Host-Parasite Interactions in the Zebra Mussel
Source: PLoS One. 2013 Jun 13;8(6):e65822. doi: 10.1371/journal.pone.0065822 (PMC3681881; doi:10.1371/journal.pone.0065822)
Supplement: File S1 — Assessment of the specificity of Hsp70 antibody (clone C92F3A5) on zebra mussel Hsp70 protein. (DOCX) [file pone.0065822.s002.docx]

**Material and methods**

**Intracellular staining**

Hemolymph was collected from pericardiac cavity and processed for antibody labeling. Briefly, hemolymph was fixed 4% Formaldehyde in PBS for 15 min at 4°C. Fixed hemocytes were washed three times in PBS-T (Tween 20, 0.1%) and permeabilized with PBS-Triton X100 0.1% for 15 min at RT. After two washes in PBS-T, hemocytes were incubated in PBS-BSA 0.5% for 10 min at 4°C. Antibodies labeling was done in PBS-BSA 0.5% with 25µg/ml of anti Hsp 70kDa-FITC (Enzo Life Sciences, Cat. No ADI-SPA-810FI) or 100 µl/ml of mouse IgG1 isotype control FITC (Thermo scientific, Cat. No SA1-12183) during 1h at 4°C. After two washes in PBS-T, hemocytes were suspended in PBS and analyzed.

**Antibody specificity analysis**

Flow cytometry analyses were done in a FACSCalibur (BD Biosciences). For each samples, 10000 events were collected in a side scatter (SSC) versus forward scatter (FSC) dot plot. FITC fluorescence was collected in FL1 channel. The same population in FSC/SSC (total hemocytes) were gated to evaluate antibody binding specificity (Figure S1-A). This gate was applied on histograms FL1/count. Acquisition and further analysis were done with CellQuestPro (BD Biosciences). The isotype control showed only really faint non specific labeling (Figure S1-B) opposite to anti Hsp 70kDa antibody which labeled clearly hemocytes (Figure S1-C). This labeling showed different intensity probably linked to subtype population in hemocytes and permeabilization efficiency.
